# Supplementary material for: Inhibitory Effects of Berberine Hydrochloride on Trichophyton mentagrophytes and the Underlying Mechanisms
Source: Molecules. 2019 Feb 19;24(4):742. doi: 10.3390/molecules24040742 (PMC6412246; doi:10.3390/molecules24040742)
Supplement: Supplementary file 1 [file molecules-24-00742-s001.zip › Supplementary file 2.pdf]

Inhibitory Effects of Berberine Hydrochloride on *Trichophyton mentagrophytes* and the Underlying Mechanisms

Chen Wen Xiao, Yan Liu, Qiang Wei, Quan An Ji, Ke Li, Li Jun Pan and Guo Lian Bao *

Institute of Animal Husbandry and Veterinary Science, Zhejiang Academy of Agricultural Sciences, Hangzhou, Zhejiang 310021, China; xiaochenwen@zaas.ac.cn (C.W.X.); liuyan@zaas.ac.cn (Y.L.); weiqrabbit@163.com (Q.W.); jiquanan@hotmail.com (Q.A.J.); like@zaas.ac.cn (K.L.); p1198600895@126.com (L.J.P.)

* Correspondence: baoguolian@zaas.ac.cn; Tel.: 86-571-8640-0373

Table qRT-PCR results correlates with illumina results.

**DGE**	**Clotrimazole**	**Control**
TR1318|c0_g1	696.99	2898.3
TR5979|c1_g1	74.308	10.622
TR10031|c19_g1	498.57	82.281
TR12215|c1_g1	3.0125	17.357
TR3885|c0_g1	765.33	3204.5
TR905|c0_g1	62.708	5.0981
**RT-PCR**	**Clotrimazole**	**Control**
TR1318|c0_g1	1.718828	7.134151
TR5979|c1_g1	664.1336	33.08873
TR10031|c19_g1	59.2662	38.147
TR12215|c1_g1	3.414851	26.59826
TR3885|c0_g1	3.378796	17.18032
TR905|c0_g1	66.06366	5.727624
**DGE**	**Berberine**	**Control**
TR1318|c0_g1	561.32	2918.4
TR12215|c1_g1	2.3085	17.498
TR7831|c2_g1	55.859	9.4938
TR3885|c0_g1	617.28	3226.7
**RT-PCR**	**Berberine**	**Control**
TR1318|c0_g1	1.63346	7.13415
TR12215|c1_g1	4.54712	26.5983
TR7831|c2_g1	12.7046	2.36789
TR3885|c0_g1	3.10613	17.1803
**DGE**	**Berberine**	**Clotrimazole**
TR5979|c1_g1	7.8593	74.925
TR905|c0_g1	13.128	63.121

RT-PCR	Berberine	Clotrimazole
TR5979|c1_g1	10.0726	664.134
TR905|c0_g1	10.956	66.0637
